# Refinement of macromolecular structures against neutron data with *SHELXL2013*

**DOI:** 10.1107/S1600576713027659

**Published:** 2013-12-07

**Authors:** Tim Gruene, Hinrich W. Hahn, Anna V. Luebben, Flora Meilleur, George M. Sheldrick

**Affiliations:** a Department of Structural Chemistry, Georg-August-University Göttingen, Tammannstrasse 4, D-37077 Göttingen, Germany; b North Carolina State University, Raleigh, NC 27695, USA; c Oak Ridge National Laboratory, Oak Ridge, TN 37831-6142, USA

**Keywords:** single-crystal neutron diffraction, macromolecular structure refinement, hydrogen restraints, *SHELXL2013*

## Abstract

Some of the improvements in *SHELX2013* make *SHELXL* convenient to use for refinement of macromolecular structures against neutron data without the support of X-ray data. The new NEUT instruction adjusts the behaviour of the SFAC instruction as well as the default bond lengths of the AFIX instructions. This work presents a protocol on how to use *SHELXL* for refinement of protein structures against neutron data. It includes restraints extending the Engh & Huber [*Acta Cryst.* (1991), A**47**, 392–400] restraints to H atoms and discusses several of the features of *SHELXL* that make the program particularly useful for the investigation of H atoms with neutron diffraction. *SHELXL2013* is already adequate for the refinement of small molecules against neutron data, but there is still room for improvement, like the introduction of chain IDs for the refinement of macromolecular structures.

## Abstract 2

*SHELXL2013* contains improvements over the previous versions that facilitate the refinement of macromolecular structures against neutron data. This article highlights several features of particular interest for this purpose and includes a list of restraints for H-atom refinement.

## 1. Introduction   

Single-crystal neutron diffraction is an experimental technique complementary to X-ray diffraction. Neutron diffraction does not change the oxidation state of ions, which is a common problem with synchrotron X-ray radiation (Hersleth & Andersson, 2011 ▶), magnetic properties can be investigated, and the strength of the interaction of neutrons with hydrogen and deuterium is of the same order of magnitude as with, for example, carbon, nitrogen or oxygen (Dianoux & Lander, 2003 ▶). For the latter reason, neutron diffraction is of particular interest for the examination of hydrogen-bond interactions between ligands and enzymes (Yamaguchi *et al.*, 2009 ▶; Blakeley *et al.*, 2008 ▶; Niimura & Bau, 2008 ▶).

To improve the data-to-parameter ratio, refinements may be performed jointly against X-ray and neutron data (Orpen *et al.*, 1978 ▶; Wlodawer & Hendrickson, 1982 ▶; Adams *et al.*, 2009 ▶). It is then important to take into account that the effective *X*—H distances for X-ray and neutron diffraction vary by nearly 20% (Allen & Bruno, 2010 ▶; Deringer *et al.*, 2012 ▶). Unfortunately, several of the 66 neutron refinements deposited in the Protein Data Bank (PDB; Berman *et al.*, 2000 ▶) appear to have been performed with *X*—H bond lengths restrained or constrained to values close to those expected for X-ray diffraction. Some of the changes in *SHELXL2013* are designed to prevent this happening by accident. Where neutron (or for that matter X-ray) data set completeness is less than 100%, restraints can be particularly valuable. *SHELXL* allows the user to place these very readily.

*SHELXL* is primarily used for small-molecule refinement but is also able to refine macromolecular structures of any size, and since the introduction of the multi-CPU version it is not much slower than other macromolecular refinement programs. It has good convergence properties because all parameters are refined simultaneously rather than in separate ‘microcycles’. In this work we show how to refine macromolecular structures against neutron data with *SHELXL2013* and we draw attention to features of *SHELXL* that make the program particularly useful for the investigation of H atoms and hydrogen bonding using neutron data.

## 2. Data   

The refinement procedure and major part of the restraints were established using the data from PDB code 3rzt, collected from a perdeuterated rubredoxin crystal (Munshi *et al.*, 2012 ▶). This data set belongs to the space group 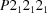 with unit-cell parameters 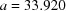, 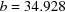, 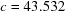
 Å. Its resolution range is 21.8–1.65 Å.

The PDB strucure 3kyx (Gardberg *et al.*, 2010 ▶) served as initial model for these data, since coordinates for 3rzt were not available at the beginning of this project.

Once consolidated, the refinement procedure was tested against the neutron data 2zoi (Yamaguchi *et al.*, 2009 ▶), the photoactive yellow protein, available from the PDB (Berman *et al.*, 2000 ▶) together with the deposited structure as initial model. 2zoi has cell dimensions 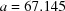, 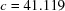
 Å in space group 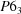 and the resolution range 33.6–1.5 Å.

## 3. Procedure   

### 3.1. Reflection data: CIF to hkl   

CIFs available from the PDB (Berman *et al.*, 2000 ▶) were converted to mtz format with *cif2mtz* (Winn *et al.*, 2011 ▶) and then converted to hkl format with *mtz2hkl* (Grune, 2008 ▶). Intensities were preferred over amplitudes whenever they were available in the deposited data.

The HKLF2 format for Laue data, in which each reflection has a different wavelength, has been available since *SHELXL97* (Sheldrick, 2008 ▶). However, while 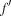 and 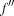 can vary strongly with wavelength for X-ray diffraction, they are zero for most elements with respect to neutron diffraction. Therefore using HKLF2 format is of no advantage unless, for example, cadmium is present in the structure, in which case wavelength-dependent scattering factors can be defined *via* the LAUE instruction.

### 3.2. Coordinates: PDB to ins   

PDB files were converted to ins format with *SHELXPRO* (Schneider & Sheldrick, 1997 ▶). H atoms belonging to water molecules were copied to the ins file; all other H atoms were removed from the ins file with the text editor *vim* (http://www.vim.org/) using regular expressions.

H atoms were placed with the default HFIX instructions generated by *SHELXPRO*. The default instruction SHEL 10 0.1 was changed to SHEL 999 0.1 to include all diffraction data. The second parameter of the CGLS was removed to include all reflections during refinement. §[Sec sec3.3]3.3 explains the consequences and justification for this step.

The NEUT instruction was placed before SFAC. This adjusts the scattering lengths according to the ILL Neutron Data Booklet (Dianoux & Lander, 2003 ▶) and also sets the default bond lengths of all HFIX and AFIX instructions as shown in Table 1 ▶.

The AFIX instruction helps with the major obstacle of neutron data: the number of H atoms approximately equals the number of all other atoms, and if they were treated like other atoms, this would double the number of parameters to be refined. H atoms placed with an AFIX instruction are constrained to limit the number of additional parameters.

When placed after the SFAC instruction(s), NEUT does not affect the interpretation of scattering lengths so that user-defined absorption coefficients can be used if required.

For perdeuterated samples, all H atoms were turned into D atoms. If, for example, H is the second and D the sixth entry of the SFAC instruction, this is achieved with the command sed "s/^H\(...\) 2/H\1 6/" name.ins > nameHtoD.ins.

Non-perdeuterated samples were checked in COOT (Emsley *et al.*, 2010 ▶) and the scattering factor manually changed from D to H for H atoms with negative nuclear density.

This procedure of starting a neutron refinement with *SHELXL* from a PDB file is realistic because it is safe to assume that an X-ray structure already exists.

The preparation was considered successful if refinement yielded 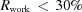 after the first round.

*SHELXPRO* automatically inserts Engh & Huber (1991 ▶) restraints in the ins file for non-H atoms of amino acids.

Restraints for water and 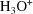 were derived from the Landolt–Börnstein series (Graner *et al.*, 1976*a* ▶,*b* ▶) and are listed in the supporting information.^1^ Isotopic differences between 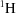 and 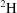 are below the number of significant digits.

The *GRADE* server (http://grade.globalphasing.org) can generate restraints for ligands including H atoms. *SHELX* format of the restraints is currently available upon request (J. Holstein, personal communication).

External restraints (Murshudov *et al.*, 2011 ▶) can be easily derived from the *ProSmart* (Nicholls *et al.*, 2012 ▶) output, for example using Awk or Perl. This can stabilize refinement against low-resolution data, especially at early stages of refinement.

### 3.3. Validation   

The *SHELXL* instruction WPDB −2 outputs a PDB file including H atoms. *Molprobity* was used to check the geometry of the refined structures (Chen *et al.*, 2010 ▶), and clashes were visually inspected in *COOT* (Emsley *et al.*, 2010 ▶). In most cases, the reported clashes needed special attention, although in some cases the clash pointed at a chemically reasonable hydrogen bond and no action was taken.

#### 3.3.1. Hydrogen restraints   

The supporting information lists restraints involving H atoms for all standard amino acids. They were validated by using these restraints instead of any AFIX constraints in the data set 2zoi (Yamaguchi *et al.*, 2009 ▶). Both variations were refined in several cycles until convergence and until the clashes reported by *Molprobity* were reduced to those which were chemically justified.

#### 3.3.2. Cross validation   

The original data set 3rzt contains only 4783 unique reflections. Proper usage of the conventional 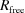 requires at least 500 reflections in the test set. Because this is a substantial part of this data set, we validated with a 50-fold cross validation: 95 hkl files were created, each with 50 reflections flagged as ‘test reflection’ for cross validation. The flags were distributed randomly in such a way that the test sets of any two hkl files were disjoint. This allows the calculation of 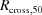, with standard deviation, instead of 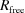. The disjoint division was created by the program *CROSSFLAGHKL*, available from TG upon request. Independence was created with the *SHELXL* instruction WIGL 0.4, which applies random shifts to the coordinates before refinement, a method also used in the *PDB_REDO* project (Joosten *et al.*, 2009 ▶). The ins file for each hkl file was created by an appropriate symbolic link to this res file after the number −1 was added to the CGLS instruction, telling *SHELXL* to exclude the reflections marked with −1 from refinement and to calculate an 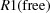 value from these reflections at the end of refinement.

## 4. Results   

In this work we tested the improvements made to *SHELXL2013* with respect to the refinement of macromolecular structures against neutron data. Our results include a set of restraints involving H atoms, listed in the supporting information, which extend the Engh & Huber (1991 ▶) restraints for amino acids. The protocol and the restraints were developed with the data set 3rzt and tested against the data set 2zoi. In both cases the models were validated using 50-fold cross validation instead of using the conventional 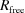. The structure for 3rzt was refined to 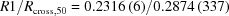 after removing one set to ensure that there were no outliers greater than 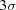 from the mean value. Interestingly, this outlier was the res file with the highest 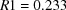 value and at the same time the lowest 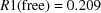 value, supporting the notion that large gaps between *R*1 and 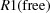 indicate model bias. The *Molprobity* clash score was reported as 18.8 and the overall *Molprobity* score was 2.07. Note that the standard deviation of 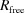 is estimated with 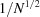 from the number of reflections in the free set (Joosten *et al.*, 2009 ▶). In order to achieve the same standard deviation of 0.0337 as calculated for full 50-fold cross validation, the size of the test set would have to be greater than 800 reflections, a large proportion of the 4784 reflections in this data set.

Since this data set served only to establish the geometry restraints and the procedure, the solvent was not fully modelled, which explains the large difference between the deposited structure and the above values: The deposited structure of 3rzt was published with 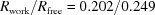, a clash score of 5.98 and an overall score of 1.33. 3kyx has 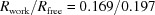, a clash score of 1.24 and an overall score of 0.84. Note that the hydrogen-bond distances present in both 3kyx and 3rzt correspond to the shortened X-ray values so that the numbers listed here are based on *Molprobity*-generated H-atom positions rather than the H-atom positions present in the deposited structures.

In contrast to the partial refinement of data set 3rzt, the data set 2zoi was fully refined. This was done in two different ways: using AFIX constraints for all H atoms except for water molecules, and with restraints instead of AFIX constraints. With AFIX constraints for H atoms, the structure refined to 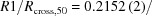 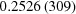. Four clear-cut outliers were ignored for the calculation of mean values and standard deviations. The *Molprobity* clash score is 5.27 and the overall score is 1.84. With 15 283 reflections, 4150 restraints and 4274 parameters, the data-to-parameter ratio is 4.55.

Replacing all AFIX commands with the restraints from this work resulted in 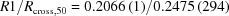 after removal of three 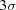 outliers, and the clash score dropped to 2.63 and the overall score to 1.53. With 15 283 reflections, 19 576 restraints and 6991 parameters, the data-to-parameter ratio is 4.99.

For comparison, the deposited structure 2zoi was published with 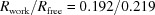, a clash score of 4.16 and an overall score of 1.75.

The following sections highlight some points of interest which make *SHELXL* particularly useful for the examination of H atoms in macromolecular structures.

### 4.1. AFIX constraints and hydrogen restraints   

The riding model cannot place all H atoms in a unique way, notably for the –OH in serine, threonine and tyrosine. These H atoms can be placed with two different AFIX instructions. Firstly one can write AFIX 87 (*i.e.* *m* = 8, *n* = 7) below the donating oxygen in the atom list and set the coordinates of the H atom in the following line to those of the accepting atom. 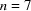 only requires the approximate direction for the placement of the H atom. Its position is then refined with the default O—H distance for 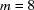 listed in Table 1 ▶ and a tetrahedral C—O—H angle.

Alternatively, when AFIX 147 is used and the coordinates of the H atom are set to 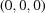, the H atom is placed by searching the circle of possible H-atom positions around the C—O bond for the minimum nuclear density for 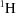 and the maximum density for 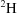. This use of AFIX 147 to place H or D attached to oxygen is best performed after the refinement of the rest of the structure, including H and D atoms, has converged. Otherwise it can be affected by difference Fourier ripples.

AFIX instructions define constraints. Unless 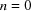, they reduce the number of refined parameters. However, since the scattering lengths of hydrogen and deuterium are significant for neutrons, the applied constraints can lead to a deterioration of both the molecular geometry and the density map. During refinement, it should therefore be tested if removing all AFIX instructions and inserting the hydrogen restraints available from the supporting information maintains the stability of the refinement.

When using AFIX it is important to bare in mind one technical difference between the riding-atom model and restrained atoms. Riding atoms experience the repulsing force of anti-bumping restraints only in the directions of the vector between the two clashing atoms. This may prevent the rotation of a group, which is often, as in the case of a methyl group, the appropriate movement to reduce a close contact violation.

The chiral volume restraint CHIV is very important for proteins, *e.g.* the chiral volume of the Cα atoms is generally restrained for all standard amino acids in macromolecular refinement. In the presence of the NEUT instruction, *SHELXL2013* distinguishes two situations: If the central atom binds to exactly three atoms, the chiral volume is calculated from these three atoms. If the central atom binds to four atoms, one of which is an H or D atom, this atom is ignored and the chiral volume relates to the remaining three atoms, thus allowing restraint of the chiral volume of Cα even in the presence of the Hα atom.

The first form allows a flexible flat amide group in proteins including the NH group, as is illustrated, for example, by Asp34 in 2zoi: the amide H atom makes a strong hydrogen bond to O in Asn38. AFIX 43 forces the H atom into the peptide plane of Asp34, which leads to a bending of the peptide plane, listed as ‘disagreeable restraint’ in the *SHELXL* listing file. If, instead of using the constraint AFIX 43, one uses CHIV_ASP N and appropriate 1,2- and 1,3-distance restraints by means of DFIX, DANG and SADI, the H atom can deviate from the peptide plane of Asp34 in a chemically plausible way.

### 4.2. Perdeuteration   

**Figure 1:**
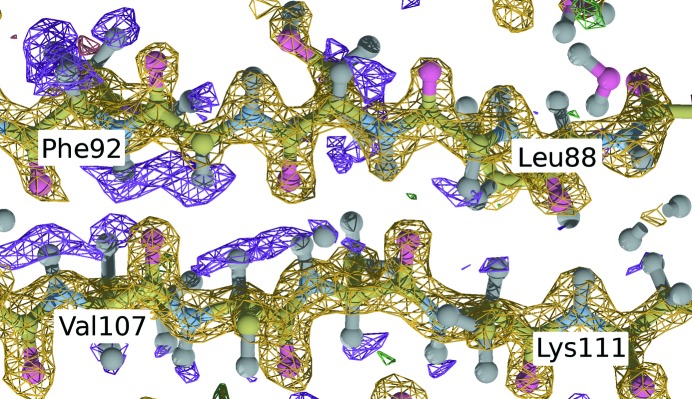
PDB code 2zoi: incomplete exchange of H atoms involved in the formation of an antiparallel β sheet. The amide H atom of Lys111 is exchanged, while those of Phe92 and Val107 are not. Yellow: 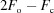 map contoured at 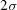; purple: 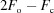 map contoured at 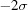.

In order to reduce background scattering from 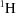, hydrogen is usually replaced with 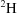 either by soaking the crystal in deuterated buffer or by perdeuteration, *i.e.* the expression of the protein in deuterated media. The exchange is nearly complete in perdeuterated samples. The ins file from *SHELXPRO* only needs adjusting once to correct the scattering factor of H atoms to deuterium as described in §[Sec sec3.2]3.2. The rubredoxin data set 3rzt was perdeuterated. When the replacement of H with D is carried out by soaking, mostly OH and NH groups exchange, while CH do not. The refinement of the non-perdeuterated sample 2zoi began with replacing all H atoms of OH and NH groups, including water molecules, with deuterium. After the first round of refinement, the nuclear density map was visually inspected in *COOT*. *COOT* has the ability to set any map as difference map, which is necessary to see the negative density values for 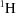 atoms. Figs. 1 ▶ and 2 ▶ show positive nuclear density in orange and negative density in purple and demonstrate how the map clearly distinguishes between hydrogen and deuterium. It is worth noting that the exchange of NH was incomplete: especially those H atoms involved in the formation of secondary structure elements were mostly unexchanged, and after our refinement, the structure of 2zoi contained 782 H atoms and only 137 D atoms in the protein chain. The antiparallel β sheet between Leu88–Phe92 and Val107–Lys111 in Fig. 1 ▶ illustrates the incomplete exchange. Note the clear positive nuclear density at the amide H atom of Lys111 and the backside of the upper strand.

### 4.3. Water molecules   

**Figure 2:**
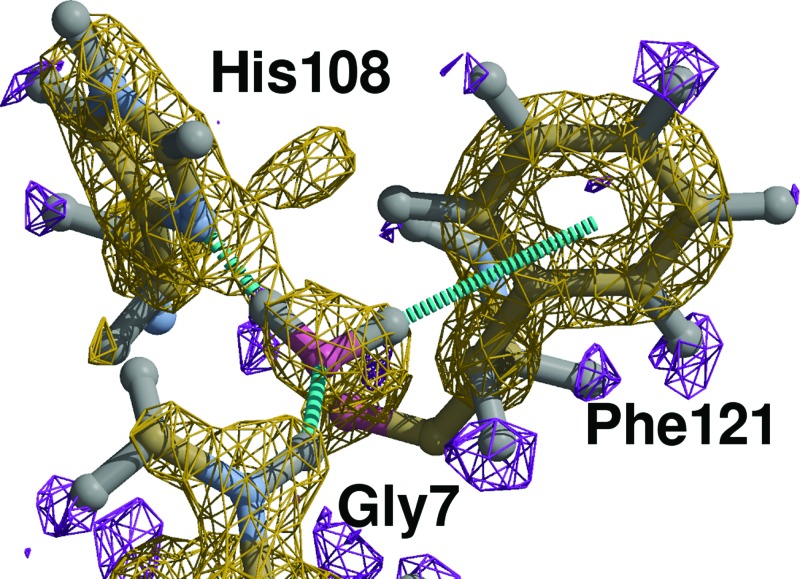
PDB code 2zoi: unrestrained orientation for a water molecule making hydrogen bonds to H atoms in Gly7 and 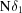 in His108. D2 points at the centre of the aromatic ring of Phe121. The hydrogen-bond network is described in §[Sec sec4.3]4.3. Yellow: 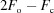 map contoured at 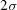; purple: 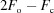 map contoured at 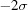. Figure created with *COOT* (Emsley *et al.*, 2010 ▶) and *Raster3d* (Merritt & Bacon, 1997 ▶).

X-ray crystallographers are used to placing water molecules as point-like O atoms. Neutron data reveal the shape of 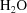, although high-resolution data are required. Fig. 2 ▶ shows one example of a water molecule oriented within the neutron data. Table 2 ▶ describes the hydrogen-bonding geometry. The position and orientation of this molecule is unrestrained. At lower resolution, a chemically reasonable orientation can be achieved by means of restraints. In the example of Fig. 2 ▶ and Table 2 ▶, where the water molecule has the residue number 2081, Gly7 the residue number 1007 and His108 the residue number 1108, simple restraints would read

DFIX 1.0 0.2 H_1007 O_2081

DFIX 1.0 0.2 D1_2081 ND1_1108

### 4.4. *SHELXL* helps to avoid mistakes   

Several of the models in the PDB refined against neutron data have a C—H bond length of 0.9 Å: the bond length used for X-ray data, which appears shortened because X-rays interact with electrons and the centre of the electron distribution lies between the donor nucleus and the H-atom nucleus. The PDB also contains several neutron data sets with occupancies refined on a per-atom basis. This leads to reduced *R* values but is difficult to justify in a chemical sense. Examples include the occupancies 0.77/0.96/0.73 of O/D1/D2 of water A2017 in PDB structure 4ar3 (Cuypers *et al.*, 2013 ▶), the occupancy −0.45 of the D atom of Val17 in 3otj (Kawamura *et al.*, 2011 ▶) and the occupancies 0.22/0.49/−0.02 for the amide group in Met1 of 3kcl (Kovalevsky *et al.*, 2010 ▶). Independent refinement of occupancies is not justified for the data-to-parameter ratio usually available for macromolecular structures.

The ‘standalone’ philosophy of *SHELXL* programs becomes also noticeable in the simplicity of input files: only the reflection data file and one single file containing coordinates, instructions and restraints are needed, and the automatically generated lst file directs the users to critical points in the model, like the list of disagreeable restraints at the end of refinement directly listed above the refinement statistics.

The concept of free variables in *SHELXL* encourages the user to inspect the occupancy values to ensure that models make chemical sense, and to recognize and avoid artefacts that lead to an artificially low *R*1 value but not to an improved structure. Free variables also allow one to create arbitrary groups of atoms in order to determine the ratio of 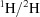 at labile positions, for example, in the solvent or for NH groups. This is explained in the following section.

### 4.5. Deuterium saturation   


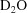
 can be considered highly hygroscopic, and the saturation with deuterium drops as soon as a buffer is exposed to the humidity of air. The data set 3rzt stems from a perdeuterated sample. To determine the D saturation of the perdeuterated rubredoxin, the occupancy of D atoms was refined initially grouped by free variables (FVAR) for

(1) N—D and O—D (including all water molecules)

(2) Cα—Dα

(3) any other C—D bond

The groups including carbon resulted in free variable values greater than 1.0. Therefore, only the occupancy of exchangeable H atoms was refined and the C—D deuterium occupancies were fixed at 1. This resulted in a free variable value 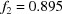. The deuterium saturation can be calculated from the total scattering contribution: 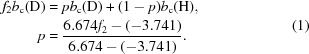

*b*_c_(H) and *b*_c_(D) are the bound coherent scattering lengths of hydrogen and deuterium, respectively (Dianoux & Lander, 2003 ▶). This results in a deuterium saturation of 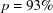, a realistic value for deuterated buffers used in perdeuteration (S. A. Mason, personal communication).

The usage of one free variable only for the occupancy of a group of D atoms introduces only one extra variable, avoids error-prone book-keeping compared to the introduction of an alternative conformation found in several deposited PDB files (*e.g.* 2zoi) and leads to the chemically useful information of an effective deuterium saturation of the sample.

### 4.6. Estimated standard deviations   

*SHELXL* has had the capability of calculating estimated standard deviations by full-matrix refinement. Since *SHELXL2013* uses dynamic memory allocation, this capability is only limited by computer RAM and is therefore also accessible to macromolecular structures. Without any further arguments, the HTAB instruction in the ins file will list putative hydrogen bonding including nonclassical hydrogen bonds in the lst file, and the res file is amended by the detailed syntax of the HTAB command. These include C—H⋯*X* bonds in the case where the C atom is attached to an electron withdrawing group. The combination of the HTAB instruction with unrestrained refinement, *i.e.* L.S. 1 DAMP 0 0 and MORE 3, provides standard deviations also for hydrogen bonding. As an example we chose 2zoi. Restraints were removed with the command sed -r "/^(DANG|DFIX|SADI|FLAT|CHIV|DELU|SIMU|BUMP)/d" 2zoi-final.res > 2zoi-esds.res.

The estimated standard deviations in the listing file include a separate table for the hydrogen-bond geometry, *e.g.* the phenol group from hydroxycinnamate in the photoactive yellow protein 2zoi. It forms a low-barrier hydrogen bond, discussed by Yamaguchi *et al.* (2009 ▶). Table 3 ▶ shows the distances and angles together with their estimated standard deviations to both Hη in Tyr42 and H∊_2_ in Glu46. Note that the hydrogen bond to Tyr42 with a short O—O distance is marked a ‘short ionic hydrogen bond’ by Yamaguchi *et al.* (2009 ▶)

Taking standard deviations into account in the discussion of hydrogen bonds may be particularly beneficial for the Hydrogen and Hydration Database (Niimura & Bau, 2008 ▶).

## 5. Conclusions   

Owing to its flexibility, *SHELXL* has always been able to refine both small-molecule and macromolecular structures, either against X-ray or against neutron data. For macromolecular structures, however, the special treatment of H atoms was an obstacle, *e.g.* the chiral volume restraint on Cα atoms was not applicable.

The NEUT instruction introduced with *SHELXL2013* overcomes many shortcomings. The short form of SFAC deploys up-to-date neutron scattering factors and absorption coefficients, making the use of *SHELXL* less prone to typing errors. H and D atoms are treated in a sensible way, allowing both their convenient treatment *via* AFIX instructions and the use of more flexible 1,2-distance (DFIX), 1,3-distance (DANG) and chiral volume (CHIV) restraints.

Thanks to the full-matrix refinement available in *SHELXL2013*, even for large structures, hydrogen bonding including estimated standard deviations can be investigated, one of the main targets for neutron diffraction of macromolecules. The possibility to include standard deviations should be beneficial for a quantitative classification of hydrogen bonds.

We are currently investigating the refinement of low-resolution data like 2efa, insulin with 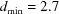
 Å (Ishikawa *et al.*, 2008 ▶). This includes the use of external restraints. The output format from *ProSmart* (Nicholls *et al.*, 2012 ▶) can be converted to *SHELXL* restraints by a single line of Awk commands and is thus particularly useful for this purpose.

## Supplementary Material

List of restraints involving H atoms. DOI: 10.1107/S1600576713027659/he5625sup1.txt

## Figures and Tables

**Table 1:** Default bond lengths in ångströms for AFIX instructions with and without the NEUT instruction for a temperature of 293 K

AFIX	*m* =	1	2	3	4	4[N]	3[N]	15[B]	8[O]	9	9[N]	16
NEUT	*X*—H	1.10	1.09	1.06	1.08	1.01	1.03	1.20	0.98	1.08	1.01	1.08
(X-ray)	*X*—H	0.98	0.97	0.96	0.93	0.86	0.89	1.10	0.82	0.93	0.86	0.93

**Table 2:** Hydrogen-bond geometry (Å, °) for the water molecule shown in Fig. 2 ▶

*D*—H⋯*A*	*D*—H	H⋯*A*	*D*⋯*A*	*D*—H⋯*A*
(Gly7) N—H ⋯O	1.01 (19)	1.8 (2)	2.7 (3)	141 (20)
O—D1⋯ND1 (His108)	1.0 (2)	1.5 (2)	2.5 (3)	175 (25)

**Table 3:** Hydrogen-bonding environment (Å, °) of O4′ in hydroxycinnamate in the structure 2zoi

		O_D_—H	H—O4′	O_D_⋯O4′	O_D_—H⋯O4′
Tyr42	Oη—Hη	1.0 (2)	1.6 (2)	2.55 (19)	160 (25)°
Glu46	O∊_2_—H∊_2_	1.16 (15)	1.66 (16)	2.78 (19)	162 (14)°

## Footnotes

1 Supporting information is available from the IUCr electronic archives (Reference: HE5625).
